# Risk assessment of venous thromboembolism and bleeding in COVID‐19 patients

**DOI:** 10.1111/crj.13467

**Published:** 2022-01-21

**Authors:** Lan Wang, Lan Zhao, Feng Li, Ji Liu, Li Zhang, Qiuhong Li, Jin Gu, Shuo Liang, Qinhua Zhao, Jinmin Liu, Jin‐fu Xu

**Affiliations:** ^1^ Department of Respirology, Shanghai Public Health Clinical Center Fudan University Shanghai China; ^2^ Department of Cardio‐Pulmonary Circulation, Shanghai Pulmonary Hospital Tongji University School of Medicine Shanghai China; ^3^ Department of Respiratory and Critical Care Medicine, Shanghai Pulmonary Hospital Tongji University School of Medicine Shanghai China; ^4^ Department of Anesthesiology, Shanghai Pulmonary Hospital Tongji University School of Medicine Shanghai China; ^5^ Department of Tuberculosis, Shanghai Pulmonary Hospital Tongji University School of Medicine Shanghai China

**Keywords:** coronavirus disease 2019, deep vein thrombosis, improve bleed risk assessment model, Padua prediction score, venous thromboembolism

## Abstract

**Background:**

The coronavirus disease 2019 (COVID‐19) is a newly recognized illness that has spread rapidly all over the world. More and more reports highlight the risk of venous thromboembolism (VTE) in COVID‐19. Our study aims to identify in‐hospital VTE risk and bleeding risk in COVID‐19 patients.

**Methods:**

We retrospectively studied 138 consecutively enrolled patients with COVID‐19 and identified in‐hospital VTE and bleeding risk by Padua Prediction Score and Improve bleed risk assessment model. The clinical data and features were analyzed in VTE patients.

**Results:**

Our findings identified that 23 (16.7%) patients with COVID‐19 were at high risk for VTE according to Padua prediction score and 9 (6.5%) patients were at high risk of bleeding for VTE prophylaxis according to Improve prediction score. Fifteen critically ill patients faced double high risk from thrombosis (Padua score more than 4 points in all 15 [100%] patients) and hemorrhage (Improve score more than 7 points in 9 [60.0%] patients). Thrombotic events were identified in four patients (2.9%) of all COVID‐19 patients. All of them were diagnosed with deep vein thrombosis by ultrasound 3 to 18 days after admission. Three (75.0%) were critically ill patients, which means that the incidence of VTE among critically ill patients was 20%. One major hemorrhage happened in critically ill patients during VTE treatment.

**Conclusion:**

Critically ill patients with COVID‐19 suffered both a high risk of thrombosis and bleeding risks. More effective VTE prevention strategies based on an individual assessment of bleeding risks were necessary for critically ill patients with COVID‐19.

**Abbreviations**
COVID‐19: coronavirus disease 2019
DVT: deep vein thrombosis
ECMO: extracorporeal membrane oxygenation
FiO_2_
: fraction of inspired oxygen
GFR: glomerular filtration rate
ICU: intensive care unit
IPC: intermittent pneumatic compression
LMWH: low molecular weight heparin
PTE: pulmonary thromboembolism
SARS‐CoV‐2: severe acute respiratory syndrome coronavirus 2
VTE: venous thromboembolism

## 1 INTRODUCTION

The coronavirus disease 2019 (COVID‐19) is a newly recognized illness that has spread rapidly around the world.^1^, ^2^, ^3^, ^4^ The clinical spectrum of COVID‐19 ranges from mild to critically ill cases. Previous studies have mentioned old age, and those with coexisting medical conditions were more likely to have a poor prognosis.^5^ These factors, as well as infection, bedridden, and respiratory failure are all risk factors for venous thromboembolism (VTE).^6^, ^7^ Once COVID‐19 is complicated with deep vein thrombosis (DVT) or fatal pulmonary thromboembolism (PTE), the treatment will be challenging, and patients with VTE may have a worse clinical outcome.

In this study, we assessed the risk of VTE and bleeding and compared the risks between critically ill patients and those of non‐critically ill in hospitalized patients with COVID‐19 from the Shanghai Public Health Clinical Center. We also report our experience with four patients that suffered clinically striking thrombotic events with COVID‐19.

## 2 METHODS

### 2.1 Study design and participants

This single‐center, retrospective, observational study was done at Shanghai Public Health Clinical Center (Shanghai, China), which is a designated hospital to treat patients with COVID‐19. We retrospectively analyzed patients from January 21, 2020, to February 21, 2020, who had been diagnosed with COVID‐19, according to WHO interim guidance.^8^ Laboratory confirmation of COVID‐19 infection was performed by the local health authority.^1^, ^2^ Critically ill patients were defined as those admitted to the intensive care unit (ICU) who required mechanical ventilation or had a fraction of inspired oxygen (FiO_2_) of at least 60% or more.^9^ Identification of critically ill patients was achieved by reviewing and analyzing admission logs and histories from all available electronic medical records and patient care resources. The Ethics Commission of Shanghai Public Health Clinical Center approved this study. Written informed consent was waived due to the rapid emergence of this infectious disease.

### 2.2 Data collection and definition of terms

Variables that were collected from the patients' electronic medical records and completed from the medical files included demographic, anthropometric, and clinical variables that are components of the Padua prediction score and Improve bleeding risk prediction score.

The Padua prediction score was calculated, for each patient at study entry, according to the weight and number of the following risk factors; active cancer (3 point), previous VTE (3 points), reduced mobility (3 points), already known thrombophilic condition (3 points), recent (≤month) trauma and/or surgery (2 points), elderly age (≥70 years) (1 point), heart and/or respiratory failure (1 point), acute myocardial infarction or ischemic stroke (1 point), acute infection and/or rheumatologic disorder (1 point), ongoing hormonal therapy (1 point), and obesity (body mass index [BMI] ≥30 kg/m^2^) (1 point). A high risk of VTE is defined as a cumulative score ≥4 and a low risk as one of <4.^10^

The Improve bleeding risk prediction score was calculated according to the weight and number of the following risk factors; active gastroduodenal ulcer (4.5 points), bleeding within past 3 months (4 points), admission platelets <50 × 10^9^ cells/L (4 points), hepatic failure (2.5 points), ICU/CCU stay (2.5 points), central venous catheter (2 points), rheumatic disease (2 points), active malignancy (2 points), age 40–80 (1.5 points), age ≥85 (3.5 points), renal disease: glomerular filtration rate (GFR) 30–59 ml/min (1 point), GFR < 30 ml/min (2.5 points). A high risk of bleeding is defined as a cumulative score ≥7 and a low risk as one of <7.^11^

The risk factors were evaluated on admission. Dynamic evaluations were further conducted if the patient's situation changed depending on the VTE and bleeding risk. Routine thromboprophylaxis was provided to patients whose Padua score more than four points or according to the clinicians' decision based on clinical presentation and D‐dimer levels, even in patients with low Padua score. For those with Improve score more than 7, intermittent pneumatic compression (IPC) or low intensive thromboprophylaxis was suggested. Lower extremity compression ultrasound (CUS) was performed for all critically ill patients and those with a high risk of VTE and a high level of D‐dimer. If possible, these patients received computed tomography pulmonary angiogram (CTPA). VTE and bleeding complications and their management were recorded.

### 2.3 Statistical analysis

Statistical calculations were done with the software package Statistical Package for the Social Sciences version 21 (IBM SPSS Statistics; Armonk, NY, USA). We expressed descriptive data as mean (standard deviation) or median (interquartile range) for continuous variables and number (%) for categorical variables. We assessed differences between critically ill patients and non‐critically ill ones using a two‐sample *t* test or Wilcoxon rank‐sum test depending on parametric or nonparametric data for continuous variables and Fisher's exact test for categorical variables. Tests were two‐sided with significance set at α less than 0.05.

## 3 RESULTS

### 3.1 Demographic and clinical characteristics

The study population included 138 hospitalized patients with confirmed COVID‐19 in Shanghai Public Health Clinical Center, of whom 15 (10.9%) were critically ill. Eighty‐one (58.7%) patients were male. The average age was 52.43 ± 16.68 years. Of the 138 patients, 56 (40.6%) had one or more coexisting medical conditions. Hypertension (39 [28.3%]) and diabetes (16 [11.6%]) were the most common coexisting conditions. Compared with non‐critically ill patients (*n* = 123), critically ill patients were significantly older (60.07 ± 14.25 years vs. 50.52 ± 15.97 years; *p* < 0.01) and were more likely to have underlying comorbidities, including atrial fibrillation (3 [20.0%] vs. 3 [2.4%]), hypertension (8 [53.3%] vs. 31 [25.2%]), and stroke (4 [26.7%] vs. 0 [0%]). Furthermore, critically ill patients had abnormally elevated D‐dimer levels (normal value: 0 ~ 0.5 μg/ml) on baseline, which was significantly higher than non‐critically ill patients (0.7 [0.4,1.4] vs. 0.4 [0.3,0.8], *p* < 0.01). There were no significant differences in gender, weight, and other complications between critically ill and non‐critically ill patients.

### 3.2 Risk prediction of VTE in COVID‐19

**FIGURE 1:**
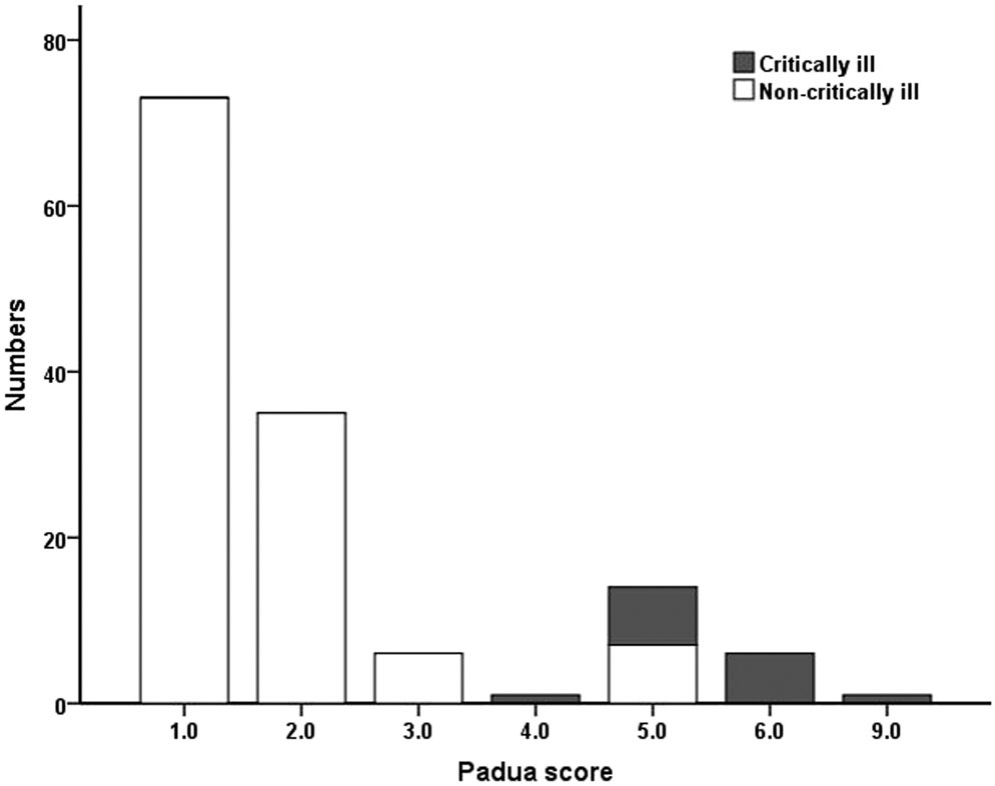
Distribution of patients and Padua scores

Padua score in COVID‐19 patients ranged from 1 point to 9 points and the median risk score was 1 point. Overall, 115 (83.3%) patients had Padua score <4 (low risk for VTE), and 23 (16.7%) patients had Padua score ≥4 (high risk for VTE). The presence of high risk for VTE was more common among patients with critically ill than those with non‐critically ill patients (15 [100%] vs. 7 [6.5], *p* < 0.01), as illustrated in Table 1 and Figure 1. The most common Padua risk parameters involved in COVID‐19 were acute infection (138 [100%]), heart failure or respiratory failure (55 [39.9%]), reduced mobility (21 [15.2%]), and elderly age (17 [12.3%]). Compared with non‐critically ill patients, critically ill patients were more likely to have the following VTE risk factors, including reduced mobility(15 [100.0%] vs. 6 [5.0%], *p* < 0.001), elderly age (7 [46.7%] vs. 10 [8.3%], *p* < 0.001), respiratory failure (11 [73.3] vs. 44 [36.4%], *p* < 0.01) and obesity (2 [13.3%] vs. 0 [0%], *p* = 0.01), as shown in Table 2. All patients at high VTE risk received adequate prophylaxis.

**TABLE 1:** Demographics and baseline characteristics of patients with COVID‐19

	All patients (*n* = 138)	Critically ill (*n* = 15)	Non‐critically ill (*n* = 123)	*p* value
Characteristics				
Age, years	52.4 ± 16.7	60.1 ± 14.3	50.5 ± 16.0	<0.01
Men, *n* (%)	81 (58.70)	12 (80.00)	69 (56.10)	0.06
Weight, kg	68.75 ± 13.71	74.33 ± 15.51	68.07 ± 13.40	0.08
D‐dimer^a^, μg/ml	0.43 (0.30,0.89)	0.74 (0.44,1.35)	0.39 (0.29,0.83)	<0.01
Comorbidity, *n* (%)				
Malignancy	4 (2.9)	1 (6.7)	3 (2.4)	0.37
Obesity	1 (0.7)	1 (6.7)	0 (0)	0.11
Liver disease	2 (1.4)	0 (0)	2 (1.6)	0.79
DM	16 (11.6)	4 (26.7)	12 (9.8)	0.08
Kidney disease	3 (2.2)	1 (6.7)	2 (1.6)	0.29
AF	6 (4.3)	3 (20.0)	3 (2.4)	0.02
CHD	7 (5.1)	2 (13.3)	5 (4.1)	0.17
Hypertension	39 (28.2)	8 (53.3)	31 (25.2)	0.03
Stroke	4 (2.9)	4 (26.7)	0 (0)	<0.001
*Padua score*				<0.001
<4, *n* (%)	115 (83.3)	0 (0)	115 (93.5)	
≥4, *n* (%)	23 (16.7)	15 (100.0)	7 (6.5)	
Improve score				<0.001
<7, *n* (%)	129 (93.5)	6 (40.0)	123 (100.0)	
≥7, *n* (%)	9 (6.5)	9 (60.0)	0 (0)	
Confirmed VTE, *n* (%)	4 (2.9)	3 (26.7)	1 (0)	<0.001
Prophylaxis, *n* (%)	41 (30.1)	15 (100.0)	26 (21.5)	<0.001

Abbreviations: AF, atrial fibrillation; CHD, coronary heart disease.

^a^ The levels of D‐dimer were obtained on admission.

**TABLE 2:** Padua prediction score component in patients with COVID‐19 on admission

	All patients (*n* = 138)	Critically ill (*n* = 15)	Non‐critically ill (*n* = 123)	*p* value
Active cancer	1 (0.7)	1 (6.7)	0 (0)	0.11
Previous VTE	1 (0.7)	15 (100.0)	1 (0.8)	0.89
Reduced mobility	21 (15.2)	15 (100.0)	6 (6.0)	<0.001
Known thrombophilic condition	0 (0)	0 (0)	0 (0)	N
Recent trauma or/and surgery	0 (0)	0 (0)	0 (0)	N
Age ≥70 years	17 (12.3)	7 (46.7)	10 (8.3)	<0.001
Heart and/or respiratory failure	55 (39.9)	11 (73.3)	44 (36.4)	0.006
Acute myocardial infarction or stroke	1 (0.7)	1 (6.7)	0 (0)	0.11
Acute infection and/or rheumatologic disorder	138 (100.0)	15 (100.0)	123 (100.0)	N
Obesity	2 (1.4)	2 (13.3)	0 (0)	0.01
Hormonal treatment	0 (0)	0 (0)	0 (0)	N

*Note*: Categorical variables are summarized with numbers and percentages.

Abbreviations: N, not applicable; VTE, venous thromboembolism.

### 3.3 Bleeding risk prediction for VTE prophylaxis in COVID‐19

**FIGURE 2:**
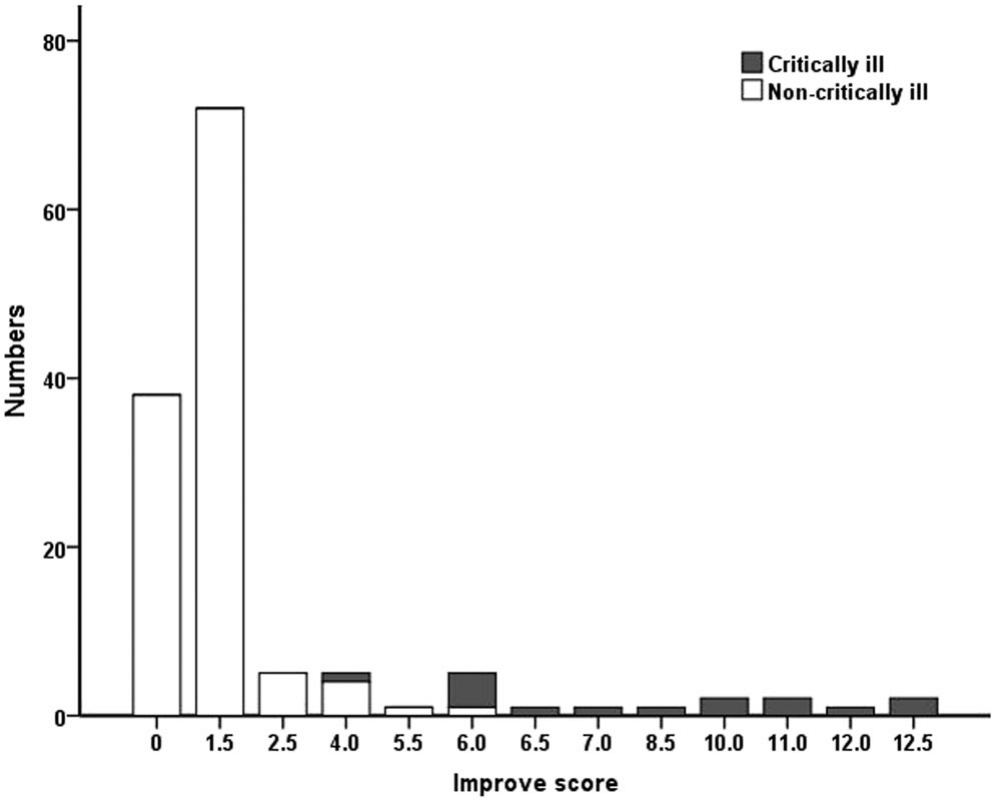
Distribution of patients and Improve scores

Improve score in COVID‐19 patients ranged from 0 point to 12.5 points and the median risk score was 1.5 point. Overall, 129 (93.5%) patients had Improve score <7 (low risk for bleeding), and 9 (6.5%) patients had Improve score ≥7 (high risk for bleeding). The presence of a high risk for bleeding was obviously more common among patients with critically ill than those with non‐critically ill patients (9 [60.0%] vs. 0 [0%], *p* < 0.01), as illustrated in Table 1 and Figure 2. The most common Improve risk parameters involved in COVID‐19 were age between 40 and 84 years (95 [68.8%]), ICU stay (15 [10.9%]), and central venous catheter (14 [10.1%]). Compared with non‐critically ill patients, critically ill patients were more likely to have the following bleeding risk factors, including bleeding with past 3 months (5 [33.3%] vs. 1 [0.8%], *p* < 0.001), hepatic failure (3 [20.0%] vs. 2 [1.6%], *p* = 0.011), ICU stay (15 [100.0%] vs. 0 [0%], *p* < 0.001), central venous catheter (13 [86.7%] vs. 1 [0.8%], *p* < 0.001), age between 40 and 84 years (14 [93.3%] vs. 81 [65.9%], *p* = 0.023), and GFR less than 30 ml/min(2 [13.3%] vs. 0 [0%], *p* = 0.011). For patients both at high risk for VTE and bleeding, most patients accepted half‐dose heparin or low molecular weight heparin (LMWH) and two patients applied IPC. A total of 6 (4.3%) patients who were all at high bleeding risk experienced bleeding event after anticoagulant therapy, including mild hematuria or microscopic hematuria (3 [2.2%]), mild gastrointestinal bleeding (1 [0.7%]), moderate nosebleed (1 [0.7%]), and severe hemothorax (1 [0.7%]) (Table 3).

**TABLE 3:** Improve prediction score component in patients with COVID‐19

	All patients (*n* = 138)	Critically ill (*n* = 15)	Non‐critically ill (*n* = 123)	*p* value
Active gastroduodenal ulcer	0 (0)	0 (0)	0 (0)	N
Bleeding within past 3 month	6 (4.3)	5 (33.3)	1 (0.8)	<0.01
Admission platelets <50 × 10^9^ cells/L	2 (1.4)	2 (13.3)	0 (0)	0.01
Hepatic failure	5 (3.6)	3 (20.0)	2 (1.6)	0.01
ICU/CCU stay	15 (10.9)	15 (100.0)	0 (0)	<0.01
Femoral catheters	14 (10.1)	13 (86.7)	1 (0.8)	<0.01
Rheumatic disease	0 (0)	0 (0)	0 (0)	N
Active malignancy	1 (0.7)	1 (6.7)	0 (0)	0.11
Age				
40–80	95 (68.8)	14 (93.3)	81 (65.9)	0.02
≥85	0 (0)	0 (0)	0 (0)	N
Renal disease				
GFR 30–59 ml/min	4 (2.9)	2 (13.3)	2 (1.6)	0.06
GFR <30 ml/min	2 (1.4)	2 (13.3)	0 (0)	0.01

*Note*: Categorical variables are summarized with numbers and percentages.

Abbreviations: GFR, glomerular filtration rate; N, not applicable.

### 3.4 Thrombotic complications in COVID‐19

Thrombotic events were identified in four patients (2.9%) of all COVID‐19 patients. All of them were diagnosed with DVT by ultrasound on the 3rd to 18th day of admission. Three (75.0%) were critically ill patients, which means that the incidence of VTE among critically ill patients was up to 20%. All four patients were male whose ages ranged from 25 to 70 years and had one or more coexisting medical conditions. Baseline D‐dimer was elevated, but to varying degrees in three patients. The other patient had a normal D‐dimer level on the baseline which increase to 3.9 μg/ml at 14 days after admission. Three of these patients received mechanical ventilation and central venous catheter treatment; one of whom used continuous renal replacement therapies (CRRT); and two had extracorporeal membrane oxygenation (ECMO) treatment. All patients accepted anticoagulant therapy with LMWH or heparin. One patient with a high risk of bleeding had severe hemothorax. All patients survived except one died of respiration failure (Table 4).

**TABLE 4:** Thrombotic complications in our patients with COVID‐19

	Patient 1	Patient 2	Patient 3	Patient 4
Age	70	25	64	64
Sex	Male	Male	Male	Male
Comorbidity	DM, HTN	Obesity	HTN	HTN
D‐dimer^a^, μg/ml	1.39	0.39	0.54	1.00
Ventilation	Y	Y	Y	N
CRRT	Y	N	N	N
ECMO	N	Y	Y	N
Venous catheterization	Y	Y	Y	N
Padua score	6	6	5	5
Improve score	5	7.5	5	1.5
VTE complication	DVT	DVT	DVT	DVT
VTE diagnosis day	3	18	8	10
Prophylaxis	LMWH	Heparin	LMWH heparin	LMWH
Bleeding event after anticoagulant therapy	None	Hemothorax	None	None
Outcome	Survived	Deceased	Survived	Survived

Abbreviations: CRRT, continuous renal replacement therapies; ECMO, extracorporeal membrane oxygenation; VTE, venous thromboembolism.

^a^ The levels of D‐dimer were obtained on admission.

## 4 DISCUSSION

Our findings identified that 16.67% of patients with COVID‐19 were at high risk for VTE according to the Padua prediction score, and 6.52% of patients were at high risk of bleeding for VTE prophylaxis according to Improve prediction score. The prediction risk of VTE (6.5%), as well as the incidence of VTE (0.8%), was low in non‐critically patients. However, critically ill patients faced double high risk from thrombosis (Padua score more than 4 points in 100% of critically ill patients) and hemorrhage (Improve score more than 7 points in 60.0% of critically ill patients). Furthermore, we identified a high incidence of VTE (20.0%) in critically ill patients with COVID‐19, despite the use of universal, guideline‐recommended thromboprophylaxis. Critically ill patients suffered a marked incidence of bleeding (26.7%), which suggested a complicated situation in VTE prophylaxis to COVID‐19.

As described, there are several reasons for the high risk of VTE in critically ill patients with COVID‐19. On the one hand, the critically ill population in our findings had three qualities in physiology, including venous stasis due to sedation or bedridden, hypercoagulability caused by glucocorticoid and immunoglobulins, and endothelial damage from venous catheterization and/or ECMO. On the other hand, a significant number of COVID‐19 patients, especially critically ill ones, were the aged^1^ who were easy to complicate with VTE high‐risk factors, such as heart failure, stroke, cancer, and diabetes. In addition, critically ill patients had a higher level of D‐dimer compared to non‐critically one,^5^ which might be associated with hypercoagulability induced by coronavirus. All these factors increased the risk of developing potentially deadly blood clots.

More interestingly, the national study in China of COVID‐19 patients had found 407 (40%) among total of 1026 patients were at high risk of VTE, which was much higher than our study.^12^ The difference might be due to two reasons. First of all, we evaluated the Padua prediction score mostly during the first week of admission. Some risk factors may be changed after 1 week, such as respiratory failure and reduced mobility. Second, the risk of reduced mobility, which had 3 points in the Padua prediction score, was low in our study. Most non‐critically ill patients had no or slight limitation during ordinary activity and were not encouraged to bed rest. No matter the different rates of COVID‐19 patients with high VTE risk, more and more attention has been paid to the role of VTE in COVID‐19.^13^, ^14^, ^15^

The COVID‐19 patients, especially critically ill ones, should pay attention to the high risk of bleeding during thromboprophylaxis. Older age is the high‐risk factor of both thrombosis and hemorrhage.^11^, ^16^ Nearly 70% of patients in our study had age‐related bleeding risk. Besides age, coexisting medical conditions, including tumors, renal or liver failure, hypertension, and diabetes, brought the risk of bleeding in our patients. Moreover, some invasive treatments increased the bleeding risk, especially ECMO, which is widely used in critically ill patients.^17^

Our findings confirmed four patients with VTE complications. The VTE rate was 2.9% in all COVID‐19 patients. Recent small‐scale investigations have shown that the incidence of VTE for COVID‐19 patients in ICU was 25% to 31%, which is much higher than the rate in our study. However, there have been few reports of VTE complicated in patients with non‐critically ill, and the total incidences of thrombotic events in COVID‐19 patients is uncertain. The recent study from Milan showed that the rate of VTE was 4.1% in COVID‐19 patients, which was similar to our study. Furthermore, the number did not mean the rate of VTE complications occurring in COVID‐19 patients was low. More likely, it was the consequence of effective thromboprophylaxis in patients classified as being at high risk of thrombosis. The high incidence of VTE in critically ill patients of COVID‐19 despite the universal use of guideline‐recommended VTE prophylaxis was similar to sepsis^18^ but markedly higher than published reports in critically ill patients without sepsis,^19^, ^20^ suggesting that dysregulated hemostasis and coagulation in severe COVID‐19.

Notably, both VTE complications and major bleeding events occurred in critically ill patients. Hence, routine thromboprophylaxis was provided to critically ill patients based on an individual assessment of their thrombosis and bleeding risks in our study. For critically ill patients with extremely high levels of D‐dimer and Fibrinogen degradation product (FDP) associated with pulmonary microthrombosis, heparin was recommended. For those at a very high risk of bleeding, mechanical prophylaxis was instituted.^21^ For those who used ECMO, better control of the aPTT (through better control of either coagulopathy or anticoagulation) was essential.^22^

This study has several limitations. First, a small population was included in this study. We hope that the findings presented here will encourage a larger cohort study. Second, this is a retrospective study. The data in this study permit a preliminary assessment of VTE and the bleeding risk of patients with COVID‐19. Further prospective studies need to determine the exact incidence of VTE among these patients and focus on hemorrhage complications during thromboprophylaxis.

In conclusion, critically ill patients with COVID‐19 suffered both a high risk of thrombosis and bleeding risks. However, the prediction risk of VTE and major bleeding was low in non‐critically patients. More effective VTE prevention strategies based on an individual assessment of bleeding risks were necessary for critically ill patients with COVID‐19.

## CONFLICT OF INTEREST

The authors declare that they have no competing interests.

## ETHICS STATEMENT

The Ethics Commission of Shanghai Public Health Clinical Center approved this study. Written informed consent was waived due to the rapid emergence of this infectious disease.

## AUTHOR CONTRIBUTIONS

Lan Wang designed the study and drafted the manuscript. Lan Zhao and Feng Li collected the data and co‐drafted the manuscript. Jinmin Liu and Jin‐fu Xu co‐designed the study and characterized patients. Ji Liu, Qiuhong Li, Jin Gu, Shuo Liang, and Qinhua Zhao characterized patients, interpreted data, and helped writing the manuscript. All authors read and approved the final manuscript.

## Data Availability

The datasets used and/or analyzed during the current study are available from the corresponding author on reasonable request.

## References

[1] Huang C , Wang Y , Li X , et al. Clinical features of patients infected with 2019 novel coronavirus in Wuhan. China Lancet. 2020;395(10223):497‐506. 31986264 10.1016/S0140-6736(20)30183-5 PMC7159299

[2] Chen N , Zhou M , Dong X , et al. Epidemiological and clinical characteristics of 99 cases of 2019 novel coronavirus pneumonia in Wuhan, China: a descriptive study. Lancet. 2020;395(10223):507‐513. 32007143 10.1016/S0140-6736(20)30211-7 PMC7135076

[3] Zhu N , Zhang D , Wang W , et al. A novel coronavirus from patients with pneumonia in China, 2019. N Engl J Med. 2020;382(8):727‐733. 31978945 10.1056/NEJMoa2001017 PMC7092803

[4] Holshue ML , DeBolt C , Lindquist S , et al. First case of 2019 novel coronavirus in the United States. N Engl J Med. 2020;382(10):929‐936. 32004427 10.1056/NEJMoa2001191 PMC7092802

[5] Yang X , Yu Y , Xu J , et al. Clinical course and outcomes of critically ill patients with SARS‐CoV‐2 pneumonia in Wuhan, China: a single‐centered, retrospective, observational study. Lancet. Respir Med. 2020;8(5):475‐481. 10.1016/S2213-2600(20)30079-5 PMC7102538 32105632

[6] Streiff MB , Agnelli G , Connors JM , et al. Guidance for the treatment of deep vein thrombosis and pulmonary embolism. J Thromb Thrombolysis. 2016;41(1):32‐67. 26780738 10.1007/s11239-015-1317-0 PMC4715858

[7] Anderson FA Jr , Spencer FA . Risk factors for venous thromboembolism. Circulation. 2003;107(23 Suppl 1):I9‐I16. 12814980 10.1161/01.CIR.0000078469.07362.E6

[8] WHO . Clinical management of severe acute respiratory infection when novel coronavirus (nCoV) infection is suspected. 2020 Jan 11, 2020.

[9] Wang D , Hu B , Hu C , et al. Clinical characteristics of 138 hospitalized patients with 2019 novel coronavirus‐infected pneumonia in Wuhan, China. Jama. 2020;323(11):1061‐1069. 32031570 10.1001/jama.2020.1585 PMC7042881

[10] Barbar S , Noventa F , Rossetto V , et al. A risk assessment model for the identification of hospitalized medical patients at risk for venous thromboembolism: the Padua Prediction Score. J Thromb Haemost. 2010;8(11):2450‐2457. 20738765 10.1111/j.1538-7836.2010.04044.x

[11] Decousus H , Tapson VF , Bergmann JF , et al. Factors at admission associated with bleeding risk in medical patients: findings from the IMPROVE investigators. Chest. 2011;139(1):69‐79. 20453069 10.1378/chest.09-3081

[12] Wang T , Chen R , Liu C , et al. Attention should be paid to venous thromboembolism prophylaxis in the management of COVID‐19. Lancet Haematol. 2020;7(5):e362‐e363. 32278361 10.1016/S2352-3026(20)30109-5 PMC7158946

[13] Klok FA , Kruip M , van der Meer NJM , et al. Incidence of thrombotic complications in critically ill ICU patients with COVID‐19. Thromb Res. 2020;191:145‐147. 32291094 10.1016/j.thromres.2020.04.013 PMC7146714

[14] Lodigiani C , Iapichino G , Carenzo L , et al. Venous and arterial thromboembolic complications in COVID‐19 patients admitted to an academic hospital in Milan. Italy Thromb Res. 2020;191:9‐14. 32353746 10.1016/j.thromres.2020.04.024 PMC7177070

[15] Zeng DX , Xu JL , Mao QX , et al. Association of Padua prediction score with in‐hospital prognosis in COVID‐19 patients. QJM. 2020;113(11):789‐793. 32652021 10.1093/qjmed/hcaa224 PMC7454846

[16] Klok FA , Barco S , Turpie AGG , et al. Predictive value of venous thromboembolism (VTE)‐BLEED to predict major bleeding and other adverse events in a practice‐based cohort of patients with VTE: results of the XALIA study. Br J Haematol. 2018;183(3):457‐465. 30123981 10.1111/bjh.15533 PMC6283241

[17] Combes A , Hajage D , Capellier G , et al. Extracorporeal membrane oxygenation for severe acute respiratory distress syndrome. N Engl J Med. 2018;378(21):1965‐1975. 29791822 10.1056/NEJMoa1800385

[18] Kaplan D , Casper TC , Elliott CG , et al. VTE incidence and risk factors in patients with severe sepsis and septic shock. Chest. 2015;148(5):1224‐1230. 26111103 10.1378/chest.15-0287 PMC4631038

[19] Cook D , Attia J , Weaver B , McDonald E , Meade M , Crowther M . Venous thromboembolic disease: an observational study in medical‐surgical intensive care unit patients. J Crit Care. 2000;15(4):127‐132. 11138871 10.1053/jcrc.2000.19224

[20] Cook D , Crowther M , Meade M , et al. Deep venous thrombosis in medical‐surgical critically ill patients: prevalence, incidence, and risk factors. Crit Care Med. 2005;33(7):1565‐1571. 16003063 10.1097/01.ccm.0000171207.95319.b2

[21] Wells PS , Forgie MA , Rodger MA . Treatment of venous thromboembolism. Jama. 2014;311(7):717‐728. 24549552 10.1001/jama.2014.65

[22] Sy E , Sklar MC , Lequier L , Fan E , Kanji HD . Anticoagulation practices and the prevalence of major bleeding, thromboembolic events, and mortality in venoarterial extracorporeal membrane oxygenation: a systematic review and meta‐analysis. J Crit Care. 2017;39:87‐96. 28237895 10.1016/j.jcrc.2017.02.014

